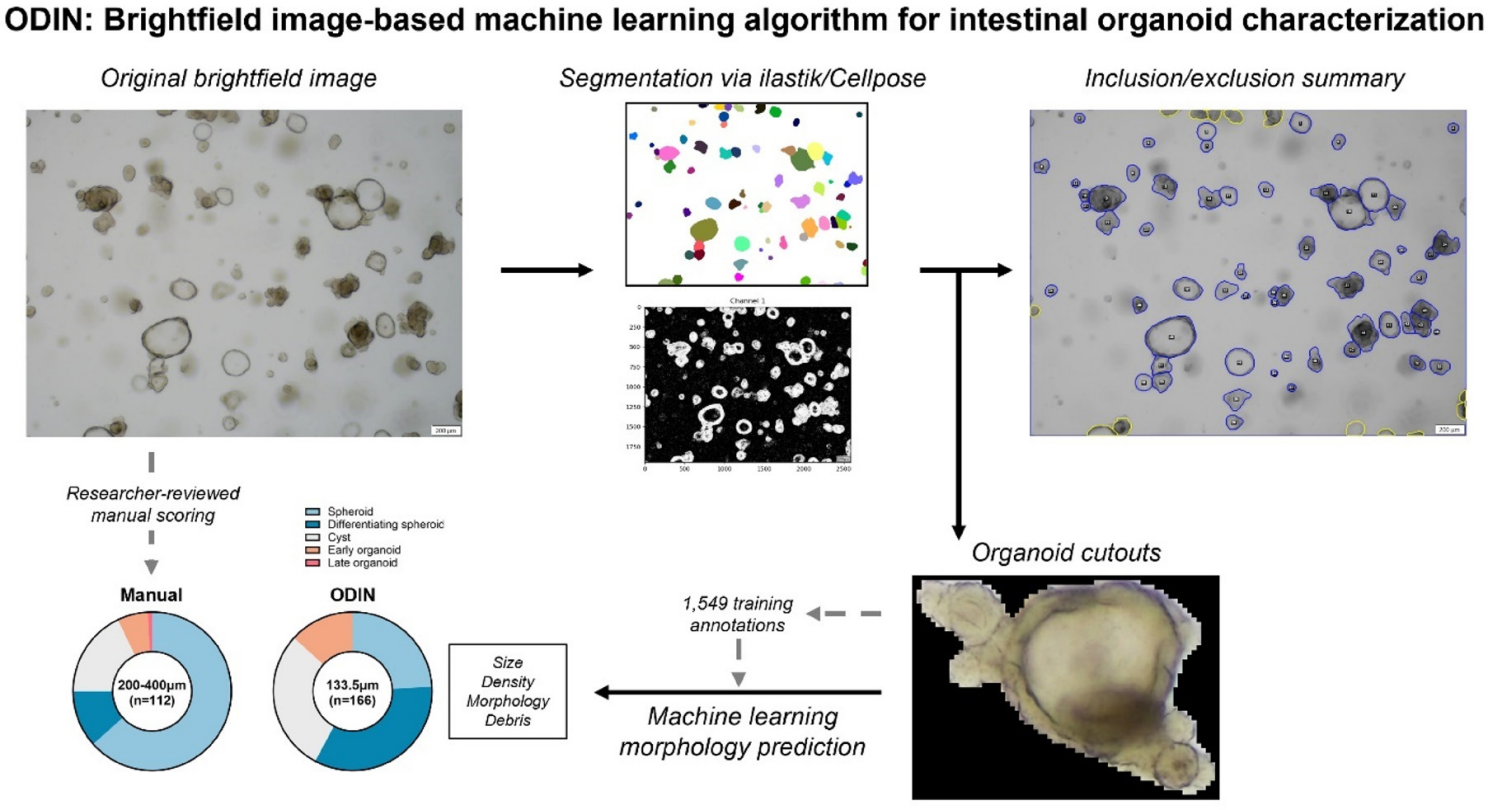# Poster Session I - Poster of Distinction I - A20 ODIN: AN UNBIASED BRIGHTFIELD IMAGE-BASED MACHINE LEARNING ALGORITHM FOR INTESTINAL ORGANOID CHARACTERIZATION

**DOI:** 10.1093/jcag/gwaf042.020

**Published:** 2026-02-13

**Authors:** H E Teslak, J Lee, S Hirota

**Affiliations:** University of Calgary, Calgary, AB, Canada; Department of Physiology and Pharmacology, University of Calgary, Calgary, AB, Canada; University of Calgary, Calgary, AB, Canada

## Abstract

**Background:**

As intestinal organoids become increasingly central to basic and translational research, standardized quality control tools are urgently needed. Current assessment methods rely heavily on manual visual inspection of brightfield microscopy images, introducing researcher bias. Existing automated image analysis tools are not optimized for the multidimensional and heterogeneous morphology of intestinal organoids. The lack of a robust, objective, and standardized evaluation method hinders the harmonization of culture quality standards and thus limiting reproducibility of organoid research.

**Aims:**

To develop a machine learning-based algorithm (ODIN) capable of analyzing brightfield organoid microscopy images and quantifying 3D culture characteristics such as quantity, size, and morphology.

**Methods:**

Generalist image segmentation tools (ilastik and Cellpose) were incorporated and modified into the ODIN workflow to aid the identification and isolation of individual intestinal organoids from brightfield images while excluding out-of-focus and overlapping organoids. A training dataset of 1,549 cutouts was manually classified by experienced organoid researchers into five morphological categories: (1) spheroid, (2) differentiating spheroid, (3) cyst, (4) early organoid, and (5) late organoid. The trained model was evaluated on five independent images not included in training, and its outputs were compared to manual annotations.

**Results:**

Across all test images, ODIN detected a greater number of individual organoids compared to manual counts, likely reflecting its ability to identify smaller or overlooked structures. Average organoid length closely approximated visual estimates but showed a slight underestimation. Morphology predictions preserved overall distribution patterns; however, spheroid and crypt-like features were respectively under- and over-classified relative to manual assessment.

**Conclusions:**

ODIN effectively segments and quantifies organoids from brightfield images, outperforming manual methods in sensitivity and required time. Over-detection of small or out-of-focus structures may contribute to size underestimation and will be addressed with further refinement. While morphology classification requires additional training data, ODIN already offers a rapid and user-friendly tool for reducing subjectivity in organoid culture assessment.

**Funding Agencies:**

Weston Family Foundation